# Cholesterol metabolism and homeostasis in the brain

**DOI:** 10.1007/s13238-014-0131-3

**Published:** 2015-02-15

**Authors:** Juan Zhang, Qiang Liu

**Affiliations:** Chinese Academy of Sciences Key Laboratory of Brain Function and Disease, and School of Life Sciences, University of Science and Technology of China, Hefei, 230026 China

**Keywords:** cholesterol metabolism, homeostasis, apoE

## Abstract

Cholesterol is an essential component for neuronal physiology not only during development stage but also in the adult life. Cholesterol metabolism in brain is independent from that in peripheral tissues due to blood-brain barrier. The content of cholesterol in brain must be accurately maintained in order to keep brain function well. Defects in brain cholesterol metabolism has been shown to be implicated in neurodegenerative diseases, such as Alzheimer’s disease (AD), Huntington’s disease (HD), Parkinson’s disease (PD), and some cognitive deficits typical of the old age. The brain contains large amount of cholesterol, but the cholesterol metabolism and its complex homeostasis regulation are currently poorly understood. This review will seek to integrate current knowledge about the brain cholesterol metabolism with molecular mechanisms.

## INTRODUCTION

Brain lipids consist of glycerophospholipids, sphingolipids, and cholesterol, they are in roughly equimolar proportions (Korade and Kenworthy, 2008). In this review, we focus on the most well studied lipid—cholesterol. Brain is the most cholesterol-rich organ, it contains about 20% of the whole body’s cholesterol (Björkhem et al., 2004). Unesterified cholesterol is the major sterol in the adult brain, and small amounts of desmosterol and cholesteryl ester are also present. The majority (about 70%–80%) of cholesterol in the adult brain is in myelin sheaths formed by oligodendrocytes to insulate axons, the rest is made up by plasma membranes of astrocytes and neurons to maintain their morphology and synaptic transmission (Dietschy and Turley, 2004).

Neurons need to build up a large amount of membrane surface of their axons, dendrites and synapses, including postsynaptic spines and presynaptic vesicles, where significantly high cholesterol content was detected (Goritz et al., 2005; Pfenninger, 2009; Takamori et al., 2006). Cholesterol is not only an essential structural component for cellular membrane and myelin, a precursor of steroid hormones and bile acid synthesis, but also a required component for synapse and dendrite formation (Goritz et al., 2005; Fester et al., 2009), axonal guidance (De Chaves et al., 1997). Cholesterol can also influence cell function through its biologically active oxidized product-oxysterol (Janowski et al., 1999; Björkhem, 2006; Radhakrishnan et al., 2007). Cholesterol is essential for neuronal physiology, both during development and in the adult stage. Cholesterol depletion in neurons impairs synaptic vesicle exocytosis, neuronal activity and neurotransmission, leads to dendritic spine and synapse degeneration (Linetti et al., 2010; Liu et al., 2010; Liu et al., 2007). Defects in cholesterol metabolism lead to structural and functional central nervous system (CNS) diseases such as Niemann-Pick C disease, Huntington’s disease, Alzheimer’s disease and Parkinson’s disease (Madra and Sturley, 2010; Block et al., 2010; Di Paolo and Kim, 2011; Wang et al., 2011).

## CHOLESTEROL SYNTHESIS AND TURNOVER IN THE BRAIN

The amount of sterol in brain ranges about 15–20 mg per g in many species, among them most of the sterol is unesterified cholesterol (Dietschy and Turley, 2004). The steady concentration remains essentially constant under normal physical conditions. However, a fraction of the pool is constantly replaced. Mechanisms must be in place to constantly excrete or degrade cholesterol, at the same time, to constantly supply an equivalent amount of new sterol to the cell plasma membranes. These two processes also must be so tightly regulated that the steady-state concentration of cholesterol in the brain remains essentially constant.

### Cholesterol synthesis in the brain

Brain cholesterol in adult mice is primarily supplied by *de novo* syntheses due to the prevention of lipoproteins uptake from the circulation by blood brain barrier (BBB) (Jeske and Dietschy, 1980). Cholesterol’s metabolism in brain is separated from the rest of the body in the presence of intact blood brain barrier. Cellular cholesterol synthesis is a complex and resource-intense process. It starts with the conversion of acetyl-CoA to 3-hydroxy-3-methylglutaryl-CoA by HMG-CoA, 3-hydroxy-3-methylglutaryl-CoA is then converted to mevalonate by HMG-CoA reductase. HMG-CoA is considered the rate-limiting and irreversible step in cholesterol synthesis. A series of enzymatic reactions occur converting mevalonate into 3-isopenenyl pyrophosphate, farnesyl pyrophosphate, squalene, lanosterol, and another 19-step process to final product-cholesterol (Berg, 2002). The majority of brain cholesterol accumulates between the perinatal period and adolescence when neurons are encircled by myelin. This *de novo* synthesis is adequate accounting for the cholesterol accumulation rate during early development, when myelin production by oligodendrocytes takes place. Moreover, the synthesis rate also closely correlates with the ultimate cholesterol level in different brain regions (Quan et al., 2003). Similar finding has been reported in rat (Jurevics and Morell, 1995; Jurevics et al., 1997). The highest cholesterol synthesis rate in human and rodents takes place when the peak of myelination process occurs, the myelination process is delayed when cholesterol synthesis is deficient (Saher et al., 2005). After myelination, the metabolism of cholesterol in the adult brain maintains at a very low turnover and minimal losses (Morell and Jurevics, 1996). The half-life of cholesterol in the adult brain is between 6 months and 5 years (Andersson et al., 1990; Björkhem et al., 2006), in contrast, the half-life of plasma cholesterol is only a few days (Dietschy and Turley, 2004).

*De novo* cholesterol synthesis takes place primarily in the endoplasmic reticulum (ER), newly synthesized cholesterol is transferred from ER to the plasma membrane (PM) rapidly (DeGrella and Simoni, 1982). The synthesis process is ATP dependent but is independent of passage through the Golgi apparatus (Kaplan and Simoni, 1985; Heino et al., 2000). The redistribution of cholesterol in different subcellular compartment is maintained by a combination of vesicle-mediated inter-organelle transport and protein-mediated monomeric transfer through the aqueous cytoplasm. Since cholesterol is water insoluble, quantitatively very little unbound cholesterol is detected in cytosol, most of the cholesterol exists as protein binding form such as apoE containing cholesterol particle in cytosol. While a role in cholesterol transport has been proposed for these proteins, whether they have additional functions other than sole transporters still remain unknown.

### Cholesterol synthesis in neurons and astrocytes

**Figure 1:**
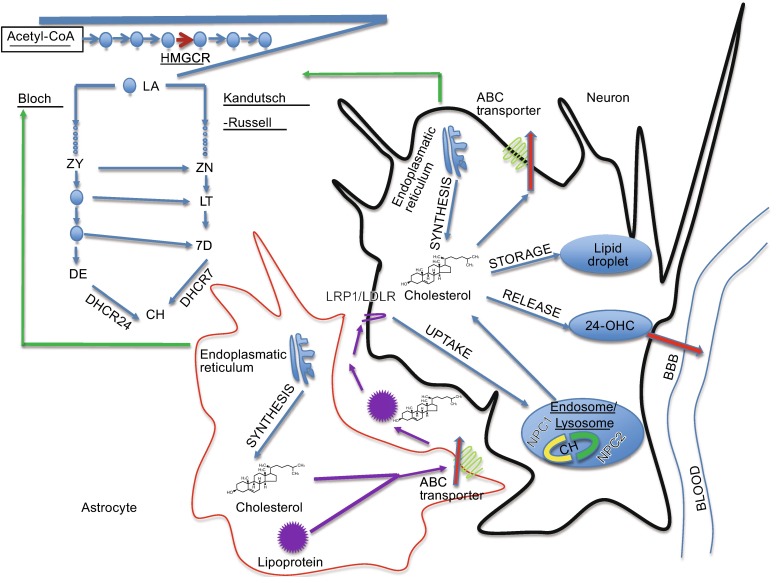
Cholesterol synthesis and metabolism in the brain. Cholesterol in neurons is primarily biosynthesized through Kandutsch-Russell pathway, whereas in astrocytes through Bloch pathway. Adult neurons essentially rely on astrocyte for cholesterol providing. Cholesterol uptake is via LRP1/LDLR receptors as apoE-containing cholesterol form. It is then converted to free cholesterol in endosome/lysosome in assistance of NPC1 and NPC2. Excess of cholesterol is prevented by intracellular esterification and storage in lipid droplets, or released as a complex with apolipoprotein-containing lipoprotein via ATP-binding cassette transporter, or converting to oxysterols then passing through BBB

Different profiles of post-squalene precursors were observed in neurons in comparison to astrocytes (Fig. 1). Neurons contain mainly sterols of Kandutsch-Russel pathway, including precursors lanosterol (LA), 7-dehydrocholesterol (7D), and lathosterol (LT) whereas astrocytes contain precursors of the Bloch pathway, such as desmosterol (DE) (Nieweg et al., 2009). In adult neurons, radioactive label was mainly found in lanosterol, whereas in glial cells it accumulated predominantly in cholesterol (Nieweg et al., 2009). A very low level of lanosterol-converting enzymes-24-dehudrocholesterol reductase (DHCR24) and lanosterol 14-alpha demethulase (CYP51) were detected in adult neurons, indicating that neurons have difficulty converting lanosterol efficiently. It was detected that adult neurons have a lower rate of sterol synthesis in comparison to glial cells. All the evidences demonstrate that adult neurons have a lower capacity to compensate for a cholesterol deficit by *de novo* synthesis in comparison to astrocytes. However, *in situ* hybridization data from the Allen Mouse Brain Atlas suggest that transcript level of many cholesterol synthesis enzymes are higher in neurons compared to these in astrocytes (Valdez et al., 2010), although higher transcript level doesn’t necessarily mean the actual protein level and enzymatic activity have the same pattern. Compartmented culture studies showed that in neurons, cholesterol synthesis is restricted to neuronal somata and does not occur in axons, but phospholipids formation takes place in both compartments, newly synthesized cholesterol in neurons is transported from soma to axon (Vance et al., 1994).

## CHOLESTEROL TURNOVER

When cholesterol synthesis rate exceeds its acquisition rate in the brain, that is when the net excretion of cholesterol occurs. Cholesterol overload often happens to adult neurons, because they primarily rely on exogenous cholesterol, whereas astrocytes produce cholesterol in excess to satisfy adult neurons’ functional needs. Several pathways for cholesterol turnover have been identified so far (Fig. 1).

### Cholesterol storage

About 1% of the total cholesterol content exists as esterified form (Bryleva et al., 2010; Liu et al., 2009), it is also called lipid droplets. This is considered as a way to store surplus cholesterol intracellularly. Cholesterol is esterified by acyl-coenzyme A: cholesterol acyltransferase 1 (ACAT1/SOAT1). This process takes place primarily in the endoplasmic reticulum. The rate of cholesterol movement from the plasma membrane to the endoplasmic reticulum is higher in cells with increased cholesterol levels, leading to an enhancement of the storage process in cells with excess unesterified cholesterol (Wustner et al., 2005). Genetic reduction of ACAT1/SOAT1 in mice decreased the concentration of cholesterol esters in the brain by 86% (Hutter-Paier et al., 2004). A genetic association study in humans also showed that a variant of ACAT1/SOAT1 gene is associated with lower levels of cholesterol in the CSF (Wollmer et al., 2003a). The ACAT1/SOAT1 enzyme is more active in neurons than in glial cells (Sakashita et al., 2000). ACAT1/SOAT1 becomes active in astrocytes under conditions like lacking apoE and when exogenous cholesterol is overloaded (Karten et al., 2006).

### Conversion to oxysterol

This is the major excretion way in the brain. The hydroxylation of cholesterol to 24-hydroxycholesterol (24-OHC) is catalyzed by cholesterol 24-hydroxylase (encoded by *CYP46A1*, which is a member of the cytochrome p450 family). Unlike non-oxydized cholesterol, oxysterols such as 24-hydroxycholesterol can cross lipophilic membranes such as the brain blood barrier (BBB) at a much faster rate than cholesterol itself (Lange et al., 1995; Meaney et al., 2002). However, the expression of this enzyme is restricted to certain types of neurons in the brain, such as pyramidal cells of the cortex and Purkinje cells of the cerebellum (Lund et al., 2003; 1999; Lütjohann et al., 1996), suggesting that these cells are particularly sensitive to excess of cholesterol. Immunocytochemical staining of cultured neurons showed that CYP46A1 is located primarily in somata and dendrites of neurons, but not in axons or presynaptic terminals (Ramirez et al., 2008). There is little expression of cholesterol 24-hydroxylase in glial cells in the brain (Ramirez et al., 2008). This indicates that the major cholesterol turnover takes place in neurons, not in astrocytes.

Disruption of the murine cholesterol 24-hydroxylase gene reduced the rate of cholesterol synthesis in the brain by 40%, whereas the brain content of cholesterol was unaltered (Lund et al., 2003), this is possibly due to the concomitant reduction in the cholesterol mevalonate pathway. All the evidences indicate that 24-hydroxylase pathway only accounts for a portion of cholesterol metabolism. Cholesterol synthesis is decreased in hippocampus of aging brain, the 24S-hydroxycholesterol level is slightly decreased as well. This could explain why the absolute cholesterol content remains at the same level (Thelen et al., 2006).

### Secretion via ABC transporter

Neurons express ABC transporters, namely ABCA1, ABCG1, and ABCG4 (Kim et al., 2008), to mediate cellular sterol efflux at the plasma membrane. ABCA1 is expressed by neurons in embryonic and adult rodents (Wellington et al., 2002; Koldamova et al., 2003), the expression level in neurons is much higher than in astrocytes (Tarr and Edwards, 2008). This pathway is CYP46A1 independent for cholesterol elimination from neurons. Cholesterol is directly released onto APOA1-containing lipoproteins that is present in CSF (Roheim et al., 1979; Pitas et al., 1987a; Koch et al., 2001), then these lipoproteins could be removed from the brain through LRP1 or scavenger receptor class B1, both of these two receptors are expressed in brain capillary endothelial cells (Panzenboeck et al., 2002; Gosselet et al., 2009). Down-regulation of ABCA1 levels in cultured neurons reduced the efflux of cholesterol onto externally supplied apoE, whereas, increased ABCA1 levels enhanced the lipid release (Minagawa et al., 2009). Taken together, neurons may handle excess cholesterol by esterification and subsequent intracellular storage, by direct excretion via ABC transporters and by conversion to 24-OHC.

## CHOLESTEROL HOMEOSTASIS IN THE BRAIN

A sufficient availability of cholesterol is necessary for normal neuronal function and morphology, neuronal cells’ function is impaired not only due to lack but also surplus of cholesterol (Ko et al., 2005; Pooler et al., 2006). Defects of cholesterol homeostasis in the adult brain are linked to neurodegenerative diseases like Niemann-Pick type C disease or Alzheimer’s disease (Madra and Sturley, 2010; Block et al., 2010; Di Paolo and Kim, 2011; Wang et al., 2011). It is well established that neuronal cells regulate their cholesterol content by an exquisite feedback mechanism that balances biosynthesis, import, and excretion. Cells sense their level of cholesterol by membrane‐bound transcription factors known as sterol regulatory element‐binding proteins (SREBPs), which regulates the transcription of genes encoding enzymes of cholesterol and fatty acid biosynthesis as well as lipoprotein receptors (Brown and Goldstein, 1999) to either increase cholesterol synthesis and uptake in sterol-depleted cells or decrease cholesterol-synthesizing enzymes when sterols are overloaded in cells (DeBose-Boyd et al., 1999; Nohturfft et al., 2000). When cholesterol reaches the maximum required level, 24-hydroxylase catalyzes cholesterol to 24-hydroxycholesterol (24-OHC), that can be eliminated in the presence of HDL as a lipid acceptor and protects neurons from the toxic effect of 24-OHC accumulation (Matsuda et al., 2013). 24-OHC, beside being a metabolite for elimination of cholesterol, it also serves as an activator of nuclear transcription factors, for example, liver X receptors α and β, which increase the expression of cholesterol transport genes (Rebeck, 2004; Tall, 2008) including ABCA1 in both neuron and glia cell (Fukumoto et al., 2002), apoE in astrocyte (Liang et al., 2004; Pfrieger and Ungerer, 2011), consequently cholesterol efflux is increased. ABCA1 is one of the major mediators for cholesterol homeostasis. During the early period of development, when the majority of growth and myelination takes place, the net cholesterol flux increase rapidly. After myelination, cholesterol synthesis continues at a very low level in the CNS. Neurons do not efficiently synthesize cholesterol after myelination completes and mainly rely on external source of cholesterol (Quan et al., 2003). Conditional ablation of cholesterol synthesis in mice neurons leads to significant transfer and uptake of glia-derived cholesterol by neurons. However, under certain condition, for example when brain-derived neurotropic factor (BDNF) is in present, the endogenous synthesis of cholesterol in neuron is partially restored (Numakawa et al., 2010). Cholesterol synthesis ablation in neuronal precursor cell during embryonic development leads reduced brain size and perinatal lethality and newly generated neurons (Saito et al., 2009). All these evidences indicate that cholesterol synthesis in neurons is essential at early development stage. On the other hand, mice lacking cellular cholesterol synthesis in adult neurons were phenotypically indistinguishable from controls, furthermore, no obivious signs of neurodegeneration or inflammation were observed (Fünfschilling et al., 2007). This indicates that cholesterol synthesis is not essential in adult neurons. Lipoprotein related protein (LRP) level remains the same in this mouse model too, this also supports that adult neurons already express sufficient LRP to import cholesterol as apoE-containing lipoprotein particles. All the evidences advocate that some adult neurons do not require cell autonomous cholesterol synthesis, which is very likely to rely on oligodendrocytes and astrocytes for cholesterol providing, especially, astrocytes, as they express apoE *in vivo* and neuronal cell can import cholesterol through receptor-mediated endocytosis of lipoproteins such as apoE binding form. The apoE-cholesterol particle is processed to free cholesterol in lysosome after being endocytosised (Ikonen, 2008; Fagan and Holtzman, 2000) and then transported to membrane. The cholesterol transport between cells is influenced by the fluidity of cell membranes and the distribution of microdomains such as lipid rafts. When membrane fluidity elevated (Xu et al., 2001), the intermolecular packing of phospholipid fatty acyl chains decreases (Ollila et al., 2007), the altered membrane composition leads to functional change.

It has been believed for a long time that glia only passively support neurons, but evidence shows that glia is actively involved in assisting neuronal functions like synaptogenesis (Pfrieger and Barres, 1997; Ullian, 2001). Cholesterol in apoE particles secreted by astrocytes increases the induced synaptic responses substantially in neuronal culture by increasing presynaptic function and dendrite differentiation (Christopherson et al., 2005; Mauch et al., 2001). Neuronal culture in presence of astrocytes showed about 10 fold more excitatory synapse activity and 5–7 fold increase of synapse numbers. Thrombospondins (TSPs), a family of extracellular matrix proteins, has found to increase synapse number in neuronal culture. Removal of TSPs from astrocyte-conditional medium diminishes the synaptogenic activity of the medium. All those indicated that TSPs are necessary and sufficient synaptogenic factor for synapse formation. Besides this, astrocytes also produce messenger RNAs that encode several synaptic adhesion proteins, including neurexins, neuroligins, and cadherins (Cahoy et al., 2008), that are known to be important for synapse formation and are believed function in neurons only (Fox and Umemori, 2006).

## MAJOR REGULATORS IN BRAIN CHOLESTEROL METABOLISM

### Apolipoproteins (apoE) in the brain

Apolipoprotein E (ApoE), a 39-kDa protein, is a major apolipoprotein in the CNS, which is highly expressed in brain, such that the brain is the organ with the second highest apoE expression after liver (Linton et al., 1991). ApoE-containing lipoproteins secreted by glial cells bind to lipoprotein receptors, and being taken up into neurons. The major function of apoE is participating in cholesterol homeostasis. Astrocytes are the major source of apoE followed by oligodendrycytes, microglia, and ependymal layer cells (Mahley et al., 2006). Neurons may express apoE under certain condition such as excitotoxic injury (Xu et al., 1999). When nerve injury happens in central nervous systems, the synthesis of apoE by glial cells increased up to 150 fold (Ignatius et al., 1986; Snipes et al., 1986; Boyles et al., 1990). There is a dynamic exchange of apoE among brain cells, as apoE is the major transport protein for extracellular cholesterol and other lipids, and that apoE-mediated cholesterol exchange occurs between neuronal and non-neuronal cells in CNS (Lahiri, 2004). The stability of apoE in the brain requires the association with lipids. The apoE level is decreased in *abca1* knockout mice, which is a gene necessary for the apoE lipidation (Wahrle et al., 2004; Hirsch-Reinshagen et al., 2005).

*In vitro* cultured astrocytes, apoE knockout showed reduced lipoprotein secretion (Piedrahita et al., 1992; Plump et al., 1992), but it remains controversial in apoE knockout mice. Some studies revealed normal cholesterol contents (Jansen et al., 2000; Lomnitski et al., 1999; Han et al., 2003) and turnover in brain, on the other hand, other studies showed reduced cholesterol level (Levi et al., 2005). Lipoproteins secreted by cultured astrocytes contain cholesterol and phospholipids, but relatively little esterified cholesterol. Some cholesterol precursors like lathosterol and desmosterol were detected in glia-derived lipoproteins, indicating that the precursor form may be converted to cholesterol in neurons after uptake. Epidemiology studies reveal the link between apoE with late-onset Alzheimer’s disease. ApoE isoform ε4 is the most common risk factor identified so far (Corder et al., 1993). ε4 alleles also correlate with amyloid plaques in Alzheimer patient’s autopsy (Schmechel et al., 1993).

### ATP-binding cassette (ABC) transporter in CNS- Lipoprotein Lipidation

ATP-binding cassette (ABC) transporters are essential component for mediating lipid transport in CNS, especially in the formation of apoE-containing lipoproteins (Tachikawa et al., 2005). ABCA and ABCG are the major classes in the brain, they are critical for lipid homeostasis (Dean et al., 2001; Schmitz et al., 2000; Puglielli et al., 2003). The core lipoprotein particle is assemblied in ER, the lipidation of nascent particles is mediated by specific subtypes of the ATP binding cassette (ABC) transporters. Cholesterol metabolite 24-OHC can up-regulate ABCA1’s expression, ABCA1, then, can mediate cholesterol efflux in the brain and influence whole-brain cholesterol homeostasis. ABCA1 catalyzes the initial transfer of lipids onto lipid-free apolipoproteins, including apoE, to form nascent particles, which are then fully lipidated in a second phase of efflux mediated by ABCG1 (Gelissen et al., 2006; Vaughan and Oram, 2006). ABCA1 is expressed in both neurons and glial cells, but much higher level in neurons than in glial cells (Wellington et al., 2002; Koldamova et al., 2003; Fukumoto et al., 2002). Neuron and glia specific ABCA1 deficiency leads to poor lipidation of apoE, and significant decrease of cholesterol level, decrease of apoE level in brain and CSF and size of apoE-containing lipoproteins in CSF (Hirsch-Reinshagen et al., 2004), suggesting that poorly lipidated apoE is more rapidly cleared. This may indicate that enhanced catabolism of apoE due to insufficient lipidation. A genetic study on human subjects showed that a single nucleotide polymorphism in the *abca1* gene highly correlates with cholesterol level in CSF (Wollmer et al., 2003b). ABCA1 transfers celluar cholesterol to acceptors like APOA1 (Oram and Heinecke, 2005). The cholesterol efflux from cultured astrocytes can be enhanced by agonists of liver x receptors (LXRs) treatment. These nuclear receptors control expression of proteins that mediate cellular cholesterol release including apoE. All the evidences indicate that ABCA1 is a crucial molecule for apoE-containing lipoprotein formation in CNS.

### LDL receptor family in the CNS

Numerous lipoprotein receptors of LDL receptor family have been identified in CNS including LDL receptor, VLDL-receptor, apoER2/LRP8, LRP4, LRP, LRP2 (megalin), LRP1B, LRP5/LRP6, and LRP11/SORL1 (Herz, 2009; Pottier et al., 2012). Ligands for these receptors are apoE-containing lipoproteins, lipids and other macromolecules (Ignatius et al., 1987; Pitas et al., 1987b). Among them, the LDL-receptor and LRP1 are the main receptors for the uptake of apoE-containing lipoprotein particles in the brain. The major difference between LRP1 and LDLR is that the latter is more highly expressed in glial cells than in neurons, but LRP1 is more highly expressed in neurons than in glial cells (Rebeck et al., 1993). LRP1 appears to have the highest transport capacity for apoE, due to its rapid endocytic rates (Li et al., 2001). ApoE associated with lipid may induce a strong anti-apoptotic effect and protect cells against neurodegeneration through an intercellular signaling pathway. LDL-receptor knockout mice have increased levels of apoE in brain parenchyma and in CSF (Liu et al., 2010), conditional deletion of *Lrp1* gene in mouse brain significantly decreases apoE, cholesterol, and sulfatide (Liu et al., 2010). ApoE lipoprotein particles secreted by glial cells have higher affinity for LDLR than LRP1, but CSF-isolated high-density lipoprotein (HDL) particles bind more strongly to LRP1 (Fagan et al., 1996). The conformation and lipidation status of apoE may affect the specificity of its receptor binding.

### NPC1/NPC2-redistribution of lipoprotein-derived cholesterol

 Externally uptaken cholesterol enters endosome/lysosome before reaches to subcellular membrane compartments (Soccio and Breslow, 2004; Storch and Xu, 2009). Two components, namely Niemann-Pick type C1 (NPC1) and C2 (NPC2), are highly involved in this process. NPC1 and NPC2 are expressed in both neurons and glial cells (Prasad et al., 2000; Ong et al., 2004; German et al., 2002; Patel et al., 1999; Hu et al., 2000). NPC1 is a transmembrane protein with a sterol-sensitive domain (Carstea et al., 1997) and NPC2 is an intralumenal component that binds cholesterol (Naureckiene et al., 2000; Soccio and Breslow, 2004). The dysfunction of either protein causes accumulation of unesterified cholesterol in the late endosome/lysosome and pathologic changes in neurons and glial cells (Reid et al., 2004; Baudry et al., 2003). Conditional NPC1 knockout in Purkinje cells leads to age-dependent motor deficits and Purkinje cell degeneration (Elrick et al., 2010), rescue of NPC1 in neurons prevented neuronal degeration (Lopez et al., 2011).

## CHOLESTEROL AND ALZHEIMER’S DISEASE

Alzheimer’s disease (AD) is a neurodegenerative disorder characterized by progressive and irreversible memory impairment and cognitive decline. The pathological hallmarks of AD are extracellular amyloid plaques of amyloid β (Aβ) peptide and intracellular neurofibrillary tangles. Cholesterol is found to be enriched in the brain plasma membranes of AD patients. The cholesterol level increases throughout the course of clinical disease, and more increase was observed when the disease progresses (Cutler et al., 2004; Xiong et al., 2008). *In vitro* study indicated that overload of cholesterol at plasma membrane in primary cultured neurons leads to an increase of Aβ production through increasing BACE1-mediated APP cleavage (Marquer et al., 2011). APP intracellular domain (AICD) release increases during this process, which down-regulates low density lipoprotein-related protein 1 (LRP1) transcription that is responsible for exogenous cholesterol capture at the plasma membrane (Liu et al., 2007), this ultimately results in a decrease of cellular cholesterol levels.

## CHOLESTEROL IN THE BRAIN AND PERIPHERAL SYSTEM

Cholesterol is an essential structural component for plasma membrane both in brain and peripheral tissue. It is required to build and maintain membrane, modulate membrane fluidity. The brain contains about 20% of whole body cholesterol, brain cholesterol is deeply involved in synapse development, synapse formation, dentrite differentiation, axonal elongation, and long-term potentiation. On the other hand, peripheral cholesterol is an important precursor molecule for the synthesis of Vitamin D and the steroid hormones. Cholesterol is converted to bile in the liver, bile salts solubilize fats and aid in intestinal absorption. Low-density lipoprotein (LDL) particles are the major blood cholesterol carriers. When LDL particles are overloaded in the blood stream, they tend to be oxidized and taken up by macrophages, which become trapped in the walls of blood vessels and contribute to atherosclerotic plaque formation. In contrast, high-density lipoprotein (HDL) particles transport cholesterol back to the liver through reverse cholesterol transport, either for excretion or for synthesis of hormones.

## CONCLUSION AND OUTLOOK

The investigation of cholesterol metabolism in the brain has a long history, but this field has gained momentum only within the last decade, probably because cholesterol is implied in neurodegenerative disease. Cells in the brain manage to keep their cholesterol content at required level in a way that is different from the rest of the body. Neurons and astrocytes, more importantly, their cooperation is essential for brain development and function. Astrocytes and neurons synthesize cholesterol by slightly different pathways and at different rates. Several pathways mediate cholesterol excretion from the brains, cholesterol hydroxylation has been proven to be the most efficient way by neurons. There is a cell-specific distribution of proteins that are involved in cholesterol metabolism. For example, apoE is highly expressed only in astrocytes, but not in neurons. This allows cholesterol transport happening between different brain cells. CYP46 is highly expressed in neurons, but not in astrocytes, this enables the removal of surplus cholesterol from the neurons.

However, questions like whether all neurons rely on cholesterol supplied by astrocytes, the regulation of cholesterol transport from astrocytes to neurons, the crosstalk between neuron and astrocyte during this process still remain unclear. The understanding of cholesterol metabolism in the brain and its role in disease requires further studies.

## Abbreviations

7D: 7-dehydro-cholesterol
24-OHC: 24-hydroxycholesterol
ABCA1: ATP-binding cassette transporter A1
AD: Alzheimer’s disease
AICD: APP intracellular domain
ApoE: apolipoprotein E
APP: Alzheimer precursor protein
BACE1: beta-secretase 1
CYP46: cytochrome P450 cholesterol 24-hydroxylase
CH: cholesterol
DE: desmosterol
DHCR7: 7-dehydrocholesterol reductase
DHCR24: 24-dehydrocholesterol reductase
HMG-CoA: 3-hydroxy-3-methylglutaryl-CoA
HMGCR: 3-hydroxy-3-methyl-glutaryl-CoA reductase
LA: lanosterol
LDLR: low density lipoprotein receptor
LRP1: low density lipoprotein receptor-related protein 1
LT: lathosterol
LXRs: liver x receptors
NPC1: Niemann-Pick type C protein 1
NPC2: Niemann-Pick type C protein 2
PD: Parkinson’s disease
ZN: zymostenol
ZY: zymosterol
